# Role of Dried Fruits of *Carissa carandas* as Anti-Inflammatory Agents and the Analysis of Phytochemical Constituents by GC-MS

**DOI:** 10.1155/2014/512369

**Published:** 2014-04-27

**Authors:** N. Anupama, G. Madhumitha, K. S. Rajesh

**Affiliations:** ^1^Chemistry Research Laboratory, Organic Chemistry Division, School of Advanced Sciences, VIT University, Vellore, Tamil Nadu 632 014, India; ^2^Department of Pharmacology, NGSM Institute of Pharmaceutical Science, Deralakatte, Mangalore 574018, India

## Abstract

Inflammation plays an important role in various diseases with high prevalence within populations such as rheumatoid arthritis, ulcer, atherosclerosis, and asthma. Many drugs are available in the market for inflammatory diseases. They exhibit several unwanted side effects to humans. Therefore, alternative treatments with safer compounds are needed. *Carissa carandas* plant is used in traditional medicinal system for its various diseases curing property. In the present study, we examined the anti-inflammatory effects of dried fruit methanol extract on carrageenan-induced hind paw edema in rats. *C. carandas* was defatted with petroleum ether, followed by methanol extraction. The methanol extracts of the dried fruits of *Carissa carandas* were given orally to the experimental rats caused significant activity (*P* ≤ 0.05) when compared with the control group. The maximum inhibition of paw edema was found to be in Group V, that is, 76.12% with inhibition of paw volume in a dose-dependent manner. The anti-inflammatory activity of the methanol extract of the dried fruits shows that the presence of potential constituents present in this extract may provide assistance in the drug discovery process. The phytochemical compounds of the extract were screened by GC-MS analysis and it was found that 11 compounds are present in methanol extract of dried fruits of *Carissa carandas*.

## 1. Introduction

Inflammation is the body's response to noxious or injurious stimuli, characterized by warmth, redness of the skin, pain, swelling, and loss of function. The response to only stimuli is quite similar. Inflammation which is a part of host defense mechanism includes several tissue factors that are known to be involved in the inflammation reactions such as release of histamines, bradykinin, and prostaglandins. Essentially, there are two types of inflammation: acute and chronic. Inflammatory diseases are mainly treated with nonsteroidal anti-inflammatory drugs and steroidal drugs [1]. During an inflammatory response, mediators such as various inflammatory mediators are released into the rat paw tissue, such as prostaglandins (PGE2), leukotrienes (LTD4), nitric oxide (NO), and oxygen reactive species. These inflammatory mediators have potential to continue stimulating the inflammatory response and its perpetuation [2]. Acute rat paw inflammation is caused by the migration of inflammatory cells into the microvascular system and fluid entering the interstitial tissue. These events are induced by inflammatory mediators which bind to specific receptors on inflammatory and endothelial cells. The first process is a sudden stiffening of these endothelial cells and inflammatory part, which occurs during the first few minutes, after an inflammatory mediator enters in bloodstream. The second event is a firm adhesion mediated by CD11/CD18 molecules and other adhesion molecules, which are necessary to maintain the inflammatory cells into the capillaries. This firm adhesion is followed by transendothelial migration into the paw tissue, leading to cell-mediated tissue injury. A variety of stimuli induce the polymorph nuclear (PMN) migration into the paw tissue. Experimental model of carrageenan-induced acute inflammation produces a range of inflammatory responses, due to the increase in the response to chemoattractants increasing PMN migration to the sites of inflammation [3].

*Carissa carandas* L. (Apocynaceae), commonly known as karaunda, is a widely used medicinal plant. The fruits, leaves, barks, and roots of* C. carandas* have been used for ethnomedicine in the treatment of human diseases, such as diarrhea, stomachic, anorexia, intermittent fever, mouth ulcer and sore throat, syphilitic pain, burning sensation, scabies, and epilepsy [4]. Chemical constituents include steroids, terpenes, tannins, flavonoid, benzenoids, phenylpropanoid, lignans, sesquiterpenes, and coumarins [5]. There are many drugs available in the market for inflammatory diseases. They exhibited several unwanted side effects to humans. Therefore, alternative treatments with safer compounds are needed. Here, the methanol extract of* Carissa carandas* dried fruits was evaluated for anti-inflammatory activity and its various phytochemical compounds were analyzed by GC-MS.

## 2. Materials and Methods

### 2.1. Plant Material

*Carissa carandas* fruits were collected from and around Vellore district, Tamil Nadu, India, during the month of October 2012. The plant specimen was authenticated and a voucher of specimen was deposited in the herbarium of Botanical Survey of India, Coimbatore (BSI/SRC/5/23/2013-14/Tech.1119). The fruits were dried in the shade at room temperature and ground to powder.

### 2.2. Preparation of the Extract

**Figure 1:**
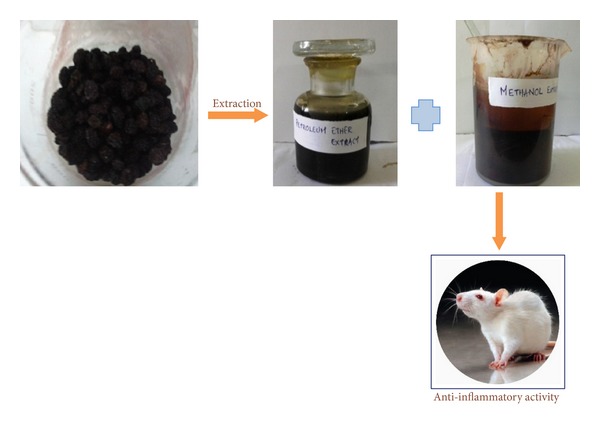
Schematic diagram of extraction and biological activity of the extract.

The powdered sample material (500 g) was defatted with petroleum ether (1000 mL). Then the residue was extracted with methanol (1000 mL) by maceration at room temperature for 48 h, stirring several times throughout the process (Figure 1). After filtering through folded paper, the methanol extract was concentrated in a rotary evaporator to yield a dark brown mass (10 g) called methanol extract (MTE). This methanol extract was subjected to GC-MS analysis and anti-inflammatory studies [6].

### 2.3. Preliminary Phytochemical Studies

The preliminary phytochemical studies were performed by using petroleum ether and methanol extract of dried fruits of* Carissa carandas* to screen the presence of various secondary metabolites [7].

### 2.4. GC-MS Analysis

The GC-MS analysis of the* C. carandas* was performed using a Clarus 680 Perkin Elmer gas chromatography equipped with an Elite-5 capillary column (5% diphenyl, 95% dimethyl polysiloxane) (30.0 m × 0.25 mmID × 250 *μ*m) and mass detector turbo mass of the company which was operated in EI mode. Helium was the carries gas used at a flow rate of 1 mL/min. The injector was operated at 200°C and the oven temperature was programmed as follows: 60°C for 2 min and 10°C/min until 300°C. Interpretation of GC-MS was conducted using the database of National Institute Standard and Technology (NIST) having more than 62,000 patterns. The spectrum of the unknown component was compared with the spectrum of the known components stored in the NIST library. The name, molecular weight, and structure of the components of the test materials were ascertained [8].

### 2.5. Pharmacological Screening

The animal experiments were performed after the approval from Institutional Animal Ethical Committee (IAEC), VIT University Vellore (VIT/IAEC/8th/33). The experiments were conducted according to the standard guidelines.

#### 2.5.1. Anti-Inflammatory Studies

*(1) Carrageenan-Induced Paw Edema.* In this experiment, a mark was made on both of the hind paws just below the tibiotarsal junction so that every time the paw could be dipped in the mercury column of plethysmograph up to the mark to ensure constant paw volume. After 30 min of the above treatment, an inflammatory edema was induced in the left hind paw by injecting 0.1 mL carrageenan (1%) in the planter tissue hind paw of all the animals. The right paw served as a reference to noninflamed paw for comparison. The initial paw volume was measured plethysmographically within 30 sec of the injection. The relative increase in the paw volume was measured in control, standard, and treated groups, 4 h after carrageenan injection. The percent increase in paw volume over the initial reading was calculated. This increase in the paw volume in animals treated with standard drug and the different doses of methanol extract of the dried fruits of* C. carandas* were compared with the increase in paw volume of untreated control animals after 3 h. The percentage inhibition of edema volume was calculated using the formula

(1) $$\begin{array}{c} \%\;\;\text{Inhibition}=\left[V_{c}-\frac{V_{t}}{V_{c}}\right]\times 100, \end{array}$$

where *V*_*t*_ and *V*_*c*_ are the relative changes in the edema of the test and control, respectively.

The results were expressed as % inhibition of edema over the untreated control group [9, 10].

*(2) Statistical Analysis.* The results were expressed as mean ± SEM. The total variation present in the data was analyzed by one-way analysis of variance (ANOVA) followed by post hoc Dunnett's test

## 3. Results and Discussion

### 3.1. Phytochemical Screening

The extracts obtained from solvent extraction with petroleum ether and methanol were then subjected to various qualitative preliminary tests for the identification of secondary metabolites. They showed the presence of alkaloids, glycosides, flavonoid, terpenes, steroids, and tannins in the methanol extract, whereas the petroleum ether extract shows only carbohydrates and steroids (Table 1).

### 3.2. GC-MS Analysis

**Figure 2:**
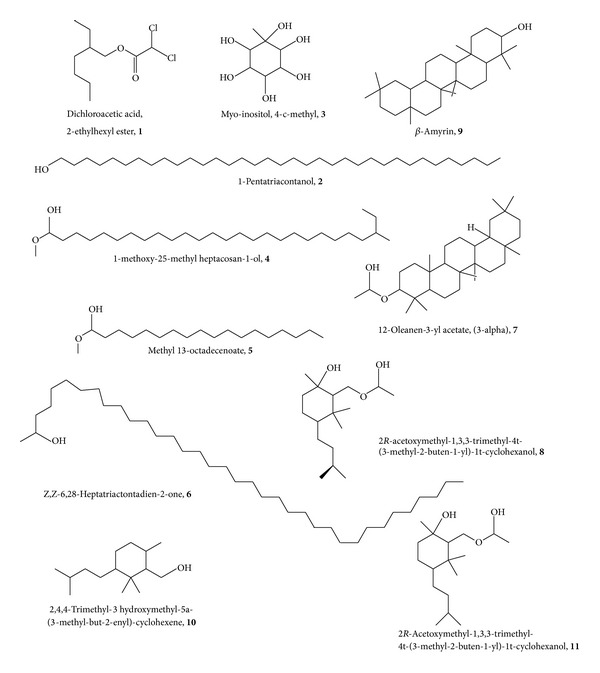
List of chemical constituents present in the extract.

Methanol extract of dried fruits of* Carissa carandas* has been analyzed by GC-MS technique. The results are given in Table 2. The extract was shown to contain a mixture of components. 11 components were identified. The analysis of methanol extract of dried fruits of* C. carandas *showed myo-inositol, 4-c-methyl (27.8%), 2*R*-acetoxymethyl-1,3,3-trimethyl-4t-(3-methyl-2-buten-1-yl)-1t-cyclohexanol (23.10), dichloroacetic acid, 2-ethylhexyl ester (15.39), 12-oleanen-3-yl acetate, (3-alpha) (10.77), other minor compounds like 1-pentatriacontanol (5.66), *β*-amyrin (2.39), Z,Z-6,28-heptatriactontadien-2-one (1.00), 1-methoxy-25-methyl heptacosan-1-ol (2.12), and 2,4,4-trimethyl-3 hydroxymethyl-5a-(3-methyl-but-2-enyl)-cyclohexene (1.63) shown in Table 2 and Figure 2.

### 3.3. Anti-Inflammatory Activity

#### 3.3.1. Carrageenan-Induced Paw Edema Method

The rats treated with oral administration of methanol extract of dried fruits of* C. carandas* reduced acute paw edema volume as compared to control. The rats were treated with dried fruit extract at a dose of 100, 200, and 400 mg/kg body weight and exhibited 33.7, 60.95, and 76.12% inhibition of paw edema volume when compared with control at 2 h, respectively. The % inhibition of paw edema increased with time and gave maximum effect at 2 h when compared with control. Here, the methanol extracts of dried fruits of* C. carandas* exhibited anti-inflammatory activity in dose-dependent manner. The values obtained from each group were expressed as mean ± SEM. To compare the statistical significant changes between control, indomethacin treated rats and with methanol extract of dried fruits of* C. carandas*, we have done Dunnett's *t*-test. The significant levels between the groups were compared between initial at varying time interval. The experiment showed (Table 3) that the extract exhibited statistically significant (*P* < 0.05) inhibition of paw volume in a dose-dependent manner. However, maximum inhibition of paw edema which was found to be in Group V is 76.12% [11].

Inflammation plays an important role in various diseases with high prevalence within populations such as rheumatoid arthritis, ulcer, atherosclerosis, and asthma. Many drugs are available in the market for inflammatory diseases. Percentage inhibition of edema volume of methanol extract and standard drug were calculated every 30 minutes up to 2 h duration. There is a dose-dependent inhibition of paw edema in rats as mentioned in Table 3. During the inflammatory response mediators like prostaglandins and bradykinins were playing an important role in carrageenan-induced paw edema. Here, in this experiment first we induced edema by injecting 1% carrageenan, thereby caused the release of autacoids, histamine, and 5-hydroxy tryptamine (5-HT). Once the inflammation starts declining from maximum, prostaglandins started to act which results in the migration of leukocytes into the inflamed site. Here, indomethacin was used as standard drug. The presence of the reported chemical constituents like myo-inositol, 4-c-methyl, 2*R*-acetoxymethyl-1,3,3-trimethyl-4t-(3-methyl-2-buten-1-yl)-1t-cyclohexanol, dichloroacetic acid, 2-ethylhexyl ester, and 12-oleanen-3-yl acetate, (3-alpha), by GC-MS analysis provided the fact that they might suppress the formation of bradykinin and prostaglandin in the system. The present study justifies the use of this plant in traditional medicine for the treatment of inflammatory diseases [12, 13].

## 4. Conclusion

Inflammatory diseases are treated by using steroidal and nonsteroidal.

Inflammatory diseases are treated by using steroidal and non-steroidal anti-inflammatory drugs that acts through COX inhibition (cyclooxygenase) enzyme can provide relief from the symptoms of inflammation and pain. Mostly these drugs are used for acute inflammation, but they failed to cure chronic inflammatory diseases, such as rheumatoid arthritis or osteoarthritis. Furthermore, these synthetic compounds exhibit several unwanted side effects to humans. Therefore, alternative treatments with safer compounds are needed. Based on the positive result from the experiment, methanol extract of dried fruits of* Carissa carandas* could potentially be used as food supplement with the purpose of providing anti-inflammatory effects as we used dried fruit extract here [14].

## Figures and Tables

**Table 1:** Phytochemical screening of petroleum ether and methanol extracts.

Chemical constituent	PET	MET
Carbohydrates	+	+
Alkaloids	−	+
Flavonoid	−	+
Tannins and phenolic	−	+
Steroids	+	+
Terpenoids	−	+

−: negative indicates the absence of the corresponding constituent, +: positive.

**Table 2:** GC-MS analysis of methanol extract of dried fruits of *Carissa carandas*.

Serial number	Retention time	Compounds	% relative abundance
1	14.93	Dichloroacetic acid, 2-ethylhexyl ester	15.39
2	16.61	1-Pentatriacontanol	5.66
3	17.12	Myo-inositol, 4-c-methyl	27.8
4	17.68	Heptacosanoic acid, 1-methoxy-25-methyl heptacosan-1-ol, methyl ester	2.12
5	19.76	Methyl 13-octadecenoate	1.25
6	23.19, 24.76	Z,Z-6,28-Heptatriactontadien-2-one	4.24
7	29.84	12-Oleanen-3-yl acetate, (3-alpha)	10.77
8	30.45	2*R*-acetoxymethyl-1,3,3-trimethyl-4t-(3-methyl-2-buten-1-yl)-1t-cyclohexanol	23.10
9	30.91	*β*-Amyrin	2.39
10	31.02	2,4,4-Trimethyl-3-hydroxymethyl-5a-(3-methyl-but-2-enyl)-cyclohexene	1.63
11	31.59	2*R*-Acetoxymethyl-1,3,3-trimethyl-4t-(3-methyl-2-buten-1-yl)-1t-cyclohexanol	8.10

**Table 3:** Anti-inflammatory activity of methanol extract of dried fruit of* Carissa carandas*.

Group	Sample	Amount of sample	Increase in paw volume (min)				% decrease in paw volume
			30	60	90	120	
I	Control	1 mL/kg	0.198 ± 0.01	0.35 ± 0.027	0.64 ± 0.021	0.77 ± 0.005	—
II	Standard	50 mg/kg	0.13 ± 0.021**	0.17 ± 0.016**	0.35 ± 0.021**	0.27 ± 0.016**	80.63
III	Methanol extract	100 mg/kg	0.21 ± 0.005**	0.29 ± 0.005**	0.48 ± 0.005**	0.57 ± 0.005**	33.7
IV	Methanol extract	200 mg/kg	0.19 ± 0.01**	0.22 ± 0.01**	0.4 ± 0.01**	0.45 ± 0.01**	60.95
V	Methanol extract	400 mg/kg	0.17 ± 0.01**	0.2 ± 0.01**	0.34 ± 0.05**	0.37 ± 0.01**	76.12

**The mean difference is significant at the 0.05 level, when compared to the control group.
